# Dystrophic Spinal Deformities in a Neurofibromatosis Type 1 Murine Model

**DOI:** 10.1371/journal.pone.0119093

**Published:** 2015-03-18

**Authors:** Steven D. Rhodes, Wei Zhang, Dalong Yang, Hao Yang, Shi Chen, Xiaohua Wu, Xiaohong Li, Xianlin Yang, Khalid S. Mohammad, Theresa A. Guise, Amanda L. Bergner, David A. Stevenson, Feng-Chun Yang

**Affiliations:** 1 Department of Anatomy and Cell Biology, Indiana University School of Medicine, Indianapolis, Indiana, United States of America; 2 Herman B. Wells Center for Pediatric Research, Indiana University School of Medicine, Indianapolis, Indiana, United States of America; 3 Department of Pediatrics, Indiana University School of Medicine, Indianapolis, Indiana, United States of America; 4 Hebei Medical University, The Third Hospital, Shijiazhuang, China; 5 Department of Endocrinology, Indiana University School of Medicine, Indianapolis, Indiana, United States of America; 6 Department of Neurology, Johns Hopkins Hospital, Baltimore, Maryland, United States of America; 7 Division of Medical Genetics, Department of Pediatrics, Stanford University, Stanford, California, United States of America; Université de Lyon - Université Jean Monnet, FRANCE

## Abstract

Despite the high prevalence and significant morbidity of spinal anomalies in neurofibromatosis type 1 (NF1), the pathogenesis of these defects remains largely unknown. Here, we present two murine models: *Nf1^flox/−^;PeriCre* and *Nf1^flox/−^;Col.2.3Cre* mice, which recapitulate spinal deformities seen in the human disease. Dynamic histomorphometry and microtomographic studies show recalcitrant bone remodeling and distorted bone microarchitecture within the vertebral spine of *Nf1^flox/−^;PeriCre* and *Nf1^flox/−^;Col2.3Cre* mice, with analogous histological features present in a human patient with dystrophic scoliosis. Intriguingly, 36–60% of *Nf1^flox/−^;PeriCre* and *Nf1^flox/−^;Col2.3Cre* mice exhibit segmental vertebral fusion anomalies with boney obliteration of the intervertebral disc (IVD). While analogous findings have not yet been reported in the NF1 patient population, we herein present two case reports of IVD defects and interarticular vertebral fusion in patients with NF1. Collectively, these data provide novel insights regarding the pathophysiology of dystrophic spinal anomalies in NF1, and provide impetus for future radiographic analyses of larger patient cohorts to determine whether IVD and vertebral fusion defects may have been previously overlooked or underreported in the NF1 patient population.

## Introduction

Neurofibromatosis type 1 (NF1) is a common autosomal dominant genetic disorder, affecting greater than two million individuals worldwide [1, 2]. Neurofibromin, the protein product of the NF1 tumor suppressor gene, functions as a guanosine triphosphatase-activating protein for Ras [3]. When mutated, haploinsufficient and/or nullizygous loss of *NF1* leads to hyperactivation of Ras signaling pathways, resulting in a wide range of malignant and nonmalignant clinical manifestations [4]. Skeletal abnormalities are frequently observed in the setting of NF1 and can include osteoporosis [5–10], increased fracture risk [11, 12], short stature [13–15], macrocephaly [14], bowing and pseudarthrosis of the long bones [16–20], chest wall deformities [20], sphenoid wing dysplasia [19, 21], and spinal deformities [22–24]. Spinal deformities in NF1 may be relatively common, with one case series reporting up to 77% of study subjects being affected [25–27].

Scoliosis is the most common spinal deformity observed in NF1 and approximately 2% of all pediatric scoliosis cases are associated with NF1 [23]. Scoliosis in NF1 can be further sub-classified as either non-dystrophic or dystrophic, based on the presence of various radiographic findings [22]. Non-dystrophic scoliosis in NF1 mimics idiopathic scoliosis in the general population, but typically presents earlier. By contrast, dystrophic scoliosis involves dysplastic osseous changes with rapid onset and progression. Characteristic radiographic features of dystrophic scoliosis include short-segment sharply angulated curves involving four to six vertebrae, vertebral rotation, vertebral wedging, scalloping of the vertebral margins, spindling of the transverse processes, pedicle defects, rib penciling, and widening of the spinal canal [22, 24]. Dystrophic scoliosis can lead to debilitating sequelae including neurological impairment due to impingement of the spinal cord. There is a risk of pseudarthrosis, or non-union, following orthopedic instrumentation of the affected vertebrae in individuals with NF1 [28–30].

Despite the high prevalence and significant morbidity associated with scoliosis and other spinal anomalies in individuals with NF1, their pathophysiology remains largely unknown. Since NF1 dystrophic scoliosis has been observed in close proximity to paraspinal plexiform neurofibromas [31, 32], it has been postulated that physical or paracrine interactions between the vertebral column and the adjacent tumor may be required to induce the pathogenesis and/or progression of dystrophic spinal deficits [33]. However, given that *Nf1* plays a pivotal role in regulating the function of multiple bone cell types including osteoclasts [34–37], mesenchymal stem cells [38], osteochondroprogenitors [39], and osteoblasts [40], the possibility that such dystrophic defects may arise *de novo* from intrinsically dysregulated bone remodeling merits further investigation.

To better understand the cellular and molecular mechanisms underlying dystrophic scoliosis in NF1, it is possible to develop animal models which accurately recapitulate the characteristic features seen in the human disease. Recently, our laboratory reported the generation of two new NF1 murine models: *Nf1* ^*flox/−*^;*PeriCre* mice, which harbor *Nf1* nullizygous mesenchymal stem cells on a systemic *Nf1* ^*+/−*^ background, and *Nf1* ^*flox/−*^;*Col2*.*3Cre* mice, which harbor conditional *Nf1* nullizygous osteoblasts on a systemic *Nf1* ^*+/−*^ background. These mice exhibit a spectrum of osseous defects including low bone mass, induced tibial fracture non-union, and runting (short stature) [41]. Cortical and trabecular bone mass was also significantly reduced in lumbar vertebrae of *Nf1* ^*flox/−*^;*Col2*.*3Cre* mice as compared to wild-type (WT) littermates [41]. Here, we extend our investigation of osseous phenotypes in *Nf1* ^*flox/−*^;*PeriCre* and *Nf1* ^*flox/−*^;*Col2*.*3Cre* mice to characterize dystrophic spinal deformities, which in part recapitulate those seen in the human disease.

## Materials and Methods

### Animals

*Nf1* ^*+/−*^ mice were obtained from Dr. Tyler Jacks at the Massachusetts Institute of Technology (Cambridge, MA) [42]. *Nf1* ^*flox/flox*^ mice were provided by Dr. Luis Parada at the University of Texas Southwestern Medical Center [43]. *PeriCre* transgenic mice were provided by Dr. Simon J. Conway at Indiana University [44], whereby Cre expression in adult MSCs is achieved under control of the 3.9kb fragment of the *periostin* promoter [41]. *Col2*.*3Cre* transgenic mice were generated as described elsewhere [45], whereby Cre expression in terminally differentiated osteoblasts is driven by the 2.3kb fragment of α1(I) collagen promoter. *Nf1* ^*flox/−*^;*PeriCre* (harboring conditional *Nf1* ^*−/−*^ MSCs on a *Nf1* ^*+/−*^ background) and *Nf1* ^*flox/−*^;*Col2*.*3Cre* mice (harboring conditional *Nf1* ^*−/−*^ osteoblasts on a *Nf1* ^*+/−*^ background) were generated by genetic intercross of *Nf1* ^*+/−*^, *Nf1* ^*flox/flox*^, *PeriCre*, and *Col2*.*3Cre* mice as described previously [41]. *Nf1* ^*flox/flox*^ (WT), *Nf1* ^*flox/−*^ (*Nf1* ^*+/−*^), and *Nf1* ^*flox/flox*^;*PeriCre* and *Col2*.*3Cre* mice were used as control. All animal studies were approved by the Indiana University Institutional Animal Care and Use Committee (#10376). Mice were euthanized by CO_2_ inhalation with cervical dislocation subsequently performed as a secondary means of ensuring death.

### Radiography

#### Mouse radiographs

Mice were anesthetized in a chamber containing an effective concentration of 1.5% isoflurane delivered in oxygen using a precision vaporizer. Whole spine anterior-posterior and lateral radiographs were acquired using a Digital Specimen Radiography (DSR) System with Biopix Software (Version No: 1.0.6., Bioptics, Inc. Tucson, AZ). Thoracic kyphosis was quantitatively assessed in lateral radiographs by applying the Cobb technique to measure the kyphotic angle between lines drawn parallel to the superior seventh thoracic (Th7) and the inferior fourth lumbar (L4) vertebral endplates [46]. In a similar fashion, Cobb angle measurement was also performed in anterior-posterior radiographs to objectively identify scoliosis and determine the severity of lateral curvature in the spine. Scoliosis was defined according to the classical definition of a Cobb angle greater than or equal to 10 degrees [47]. Vertebral wedging was defined quantitatively by the angle formed by the intersection of lines drawn parallel to the rostral and caudal vertebral end-plates as described by Funasaki and colleagues [48].

#### Patient radiographs

Radiographs of individuals with NF1 with features of dystrophic scoliosis were obtained from the University of Utah NF Center (Salt Lake City, UT) and Johns Hopkins Comprehensive NF Center (Baltimore, MD). Human subject research was performed in accordance with approval from the Indiana University IRB (#1011003777), the University of Utah IRB (#00011143), and the Johns Hopkins University IRB (#NA_00016949). Patient radiographs were analyzed anonymously. Nonetheless, verbal and written informed consent was obtained prior to publication of medical images.

### Micro-computed tomography (μCT)

#### Murine samples

Bone volume and microarchitecture of dystrophic vertebrae from *Nf1* ^*flox/−*^;*Col2*.*3Cre* mice were evaluated using a high-resolution desktop micro-computed tomography imaging system (μCT-20; Scanco Medical AG, Bassersdorf, Switzerland). Corresponding vertebral segments in WT mice were evaluated as a control. Specimens were scanned with a slice increment of 9 μm. μCT images were reconstructed, filtered (σ = 0.8 and support = 1.0), and thresholded (22% of maximum possible gray scale value). Trabecular bone was outlined, excluding the cortical and subcortical bone. Every 10 sections were contoured manually, and the intermediate sections were interpolated with the contouring algorithm to create a volume of interest. Microarchitecture parameters were computed including bone volume fraction (BV/TV, %), trabecular number (Tb.N, mm^−1^), trabecular thickness (Tb.Th, μm), and trabecular spacing (Tb.Sp, μm).

#### Human samples

The T5 facet of an individual with NF1 with dystrophic scoliosis and the T7 facet of an individual with scoliosis without NF1 were scanned in the gantry of a VivaCT 40 micro-computed tomographer (Scanco Medical AG, Bassersdorf, Switzerland). Images were acquired at a voxel size of 10.5 μm. Images were acquired at a voxel size of 10.5 μm. The voxel of interest (VOI) comprised the whole bone excluding the cortical margins. Parameters of bone microarchitecture including BV/TV, Tb.N, Tb.Th, Tb.Sp, and connectivity density (Conn.D., mm^−3^) were calculated. Human subject research was performed in accordance with approval from the Indiana University IRB (#1011003777) and the University of Utah IRB (#00011143). Data from human bone specimens were analyzed anonymously. Nonetheless, verbal and written informed consent was obtained prior to publication of these data.

### Histology

#### Murine samples

Mice with dystrophic vertebral defects were sacrificed at various timepoints including 1, 2, 6, 9, and 12-months of age. The dystrophic vertebral segments were dissected with corresponding segments harvested from age matched WT mice as the control. 4-μm-thick sections were cut in the sagittal plane, and stained with hematoxylin and eosin (H&E). Slides were examined at 100x magnification on a Leitz DMRXE microscope (Leica Microsystems, Wetzlar, Germany). Images were captured using a SPOT digital camera (Diagnostic Instruments, Inc., Sterling Heights, MI).

#### Human samples

The T5 facet of an individual with NF1 with dystrophic scoliosis and the T7 facet of an individual with scoliosis without NF1 were fixed in 10% formalin for 48 hours, decalcified in 10% EDTA for 2 weeks, and cut to 3.5 μm thick longitudinal sections using a rotary microtome (Leica Microsystems, Wetzlar Germany). Sections were stained for H&E and imaged using a Leitz DMRXE microscope (Leica Microsystems, Wetzlar, Germany) equipped with a SPOT digital camera (Diagnostic Instruments, Inc., Sterling Heights, MI). Human subject research was performed in accordance with approval from the Indiana University IRB (#1011003777) and the University of Utah IRB (#00011143). Data from human bone specimens were analyzed anonymously. Nonetheless, verbal and written informed consent was obtained prior to publication of these data.

### Dynamic histomorphometry

Fluorochrome labeling of the bones was performed in 4 week-old mice by intraperitoneal injections of calcein (20 mg/kg, Sigma Chemical, St. Louis, MO, USA) and alizarin red (20 mg/kg, Sigma) 8 and 3 days prior to euthanasia. Following euthanasia, L5 vertebrae were fixed in 10% neutral buffered formalin for 48 hours, dehydrated in graded ethanols, and embedded undecalcified in methylmethacrylate. Mid-sagittal sections (4 μm thick) were cut from the vertebrae. Sections were examined at 200x magnification on a Leitz DMRXE microscope (Leica Microsystems, Wetzlar, Germany). Images were captured using a SPOT digital camera (Diagnostic Instruments, Inc., Sterling Heights, MI). The region of interest was established by a boundary beginning 0.5 mm proximal to the midpoint of the growth plate, non-inclusive of cortical bone, and extending proximally for a total area of approximately 2.8 mm^2^. Trabecular bone turnover was assessed by measuring the extent of single label (sL.Pm), double label (dL.Pm) and the area of bone (dL.Ar) between the calcein and alizarin labels using Image Pro Plus version 4.1 software (Media Cybernetics, Silver Spring, MD). Derived histomorphometric parameters included mineralizing surface (MS/BS, %), a measure of active bone-forming surface, calculated as follows: the [^1^/_2_ sL.Pm + dL.Pm]/Tt.Pm * 100; mineral apposition rate (MAR, μm/day), a measure of the rate of radial expansion of new bone, calculated as follows: dL.Ar/dL.Pm/4 dy; and bone formation rate (BFR), an overall measure of bone formation that combines MS/BS and MAR, calculated as follows: MS/BS * MAR * 3.65.

### Statistical analysis

Differences between experimental groups were determined by the Student’s t-test or one-way analysis of variance (ANOVA), followed by Newman-Keuls multiple comparison tests where appropriate. *P* values < 0.05 were considered significant.

## Results

### Animal Data

#### *Nf1* ^*flox/−*^;*PeriCre* and *Col2*.*3Cre* transgenic mice recapitulate hallmark clinical features of NF1 dystrophic kyphoscoliosis

Scoliosis is defined clinically as an abnormal lateral curvature of the spine with a Cobb angle greater than or equal to 10 degrees [47]. Applying this clinical criterion to the NF1 murine model, a series of radiographs acquired from WT, *Nf1* ^*+/−*^, *Nf1* ^*flox/flox*^;*PeriCre* and *Nf1* ^*flox/−*^;*PeriCre* transgenic mice were examined in a blinded fashion and screened for the presence of scoliosis using the Cobb method [47]. We observed no evidence of scoliotic deformities in WT or *Nf1* ^*+/−*^ mice; however, 3/43 (∼7%) *Nf1* ^*flox/flox*^;*PeriCre* and 15/41 (∼37%) *Nf1* ^*flox/−*^;*PeriCre* animals exhibited radiographic evidence of scoliosis (Table 1). Moreover, the mean Cobb angle in *Nf1* ^*flox/flox*^;*PeriCre* mice with scoliotic deformities was approximately 11 degrees, versus 16 degrees in *Nf1* ^*flox/−*^;*PeriCre* mice, with three out of 15 animals exhibiting curvatures of greater than 20 degrees. Consistent with these findings, *Nf1* ^*flox/flox*^;*Col2*.*3Cre* and *Nf1* ^*flox/−*^;*Col2*.*3Cre* mice exhibit a similar phenotype, with an incidence of scoliotic deformities of ∼55% in *Nf1* ^*flox/−*^;*Col2*.*3Cre* mice as compared to ∼20% in *Nf1* ^*flox/flox*^;*Col2*.*3Cre* animals. Collectively, the increase penetrance of scoliotic deformities in *Nf1* ^*flox/−*^;*PeriCre* and *Nf1* ^*flox/−*^;*Col2*.*3Cre* mice suggests that *Nf1* heterozygosity in at least of subset of cell lineages together with biallelic *Nf1* inactivation in MSCs/osteoblasts play a critical role in the pathogenesis of scoliosis in theses NF1 murine models.

**Table 1:** Frequency of scoliotic deformities in *Nf1* ^*flox/flox*^ and *Nf1* ^*flox/−*^;*PeriCre* and *Col2*.*3* mice.

Genotype	*n*	Scoliosis present	% Affected	Mean Cobb Angle (± SD)
WT	29	0	0.00	-
*Nf1* ^*+/−*^	26	0	0.00	-
*Nf1* ^*flox/flox*^;*PeriCre*	43	(3/43)	6.98	11.48 ± 1.24
*Nf1* ^*flox/−*^;*PeriCre*	41	(15/41)	36.59	16.01 ± 5.12
*Nf1* ^*flox/flox*^;*Col2*.*3Cre*	15	(3/15)	20.0	16.46 ± 9.65
*Nf1* ^*flox/−*^;*Col2*.*3Cre*	20	(11/20)	55.0	16.60 ± 5.52

Cohorts of WT (n = 29), *Nf1* ^*+/−*^ (n = 26), *Nf1* ^*flox/flox*^;*PeriCre* (n = 43), *Nf1* ^*flox/−*^;*PeriCre* (n = 41), *Nf1* ^*flox/flox*^;*Col2*.*3Cre* (n = 15), and *Nf1* ^*flox/−*^;*Col2*.*3Cre* (n = 20) mice were radiographed in the anterior-posterior position and screened for the presence of scoliosis. The frequency (%) of mice affected and the mean Cobb angle (± standard deviation (SD)) are summarized in the table as shown.

**Fig 1:**
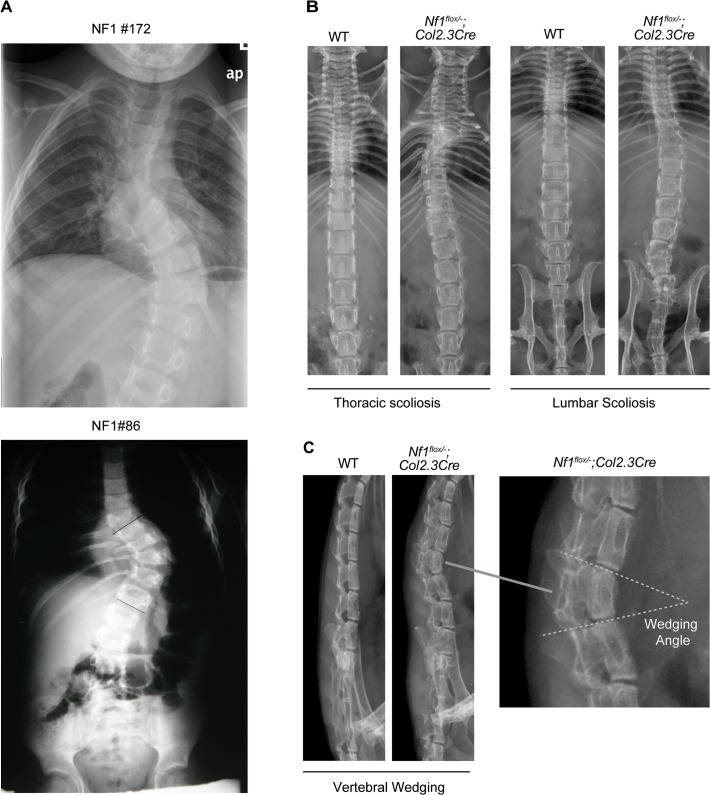
*Nf1* ^flox/−^;*Col2*.*3Cre* mice exhibit characteristic features of NF1 dystrophic scoliosis. (**A**) Anterior-posterior radiographs of two NF1 patients with dystrophic scoliosis involving the thoracic (top) and lumbar spine (bottom). (**B**) Anterior-posterior radiographs demonstrate representative cases of short-segmented scoliotic deformities in *Nf1* ^*flox/−*^;*Col2*.*3Cre* mice involving the thoracic (second panel) and lumbar spine (fourth panel). Significant vertebral rotation is further evident in the representative *Nf1* ^*flox/−*^;*Col2*.*3Cre* mouse with thoracic scoliosis as shown (second panel). (**C**) Vertebral wedging, another hallmark feature of NF1 dystrophic scoliosis, is illustrated in representative lateral radiographs of the lumbar spine. The wedging angle was measured by the intersection of lines drawn parallel to the rostral and caudal vertebral endplates as shown.

Characteristic radiographic features of NF1 dystrophic scoliosis include short-segment curves involving four to six vertebrae, vertebral rotation, vertebral wedging, scalloping of the vertebral margins, spindling of the transverse processes, pedicle defects, rib penciling, and widening of the spinal canal [22, 24]. Here we provide radiographic evidence demonstrating the presence of several of these hallmark dystrophic features in *Nf1* ^*flox/−*^;*Col2*.*3Cre* mice with correlates with the human disease. Representative radiographs illustrate short-segment, sharply angulated curves of the thoracic and lumbar spine in two individuals with a clinical diagnosis of NF1 (Fig. 1A). In comparison, we observed similar short-segment curves in the thoracic and lumbar spine of *Nf1* ^*flox/−*^;*Col2*.*3Cre* mice (Fig. 1B). Significant vertebral rotation, another hallmark dystrophic feature, is also apparent in this representative radiograph of a *Nf1* ^*flox/−*^;*Col2*.*3Cre* mouse with thoracic kyphoscoliosis (Fig. 1B, second panel).

We additionally documented the presence of vertebral wedging in three of 20 *Nf1* ^*flox/−*^;*Col2*.*3Cre* mice (Fig. 1C), with a mean wedging angle of 31.32 ± 7.15 (SD) degrees. Vertebral wedging was also observed in three of 41 *Nf1* ^*flox/−*^;*PeriCre* and one of 45 *Nf1* ^*flox/−*^;*PeriCre* animals, with a mean wedging angle of 32.55 ± 4.72 (SD) degrees in the *Nf1* ^*flox/−*^;*PeriCre* cohort. This phenotype was not apparent in the other control genotypes including *Nf1* ^*flox/flox*^;*Col2*.*3Cre* (n = 15), *Nf1* ^*+/−*^ (n = 26), and WT (n = 29) mice. Other dystrophic characteristics including spindling of the transverse processes, pedicle defects, and rib penciling were not radiographically apparent in either the *Nf1* ^*flox/−*^;*Col2*.*3Cre* or *PeriCre* mouse models.

**Fig 2:**
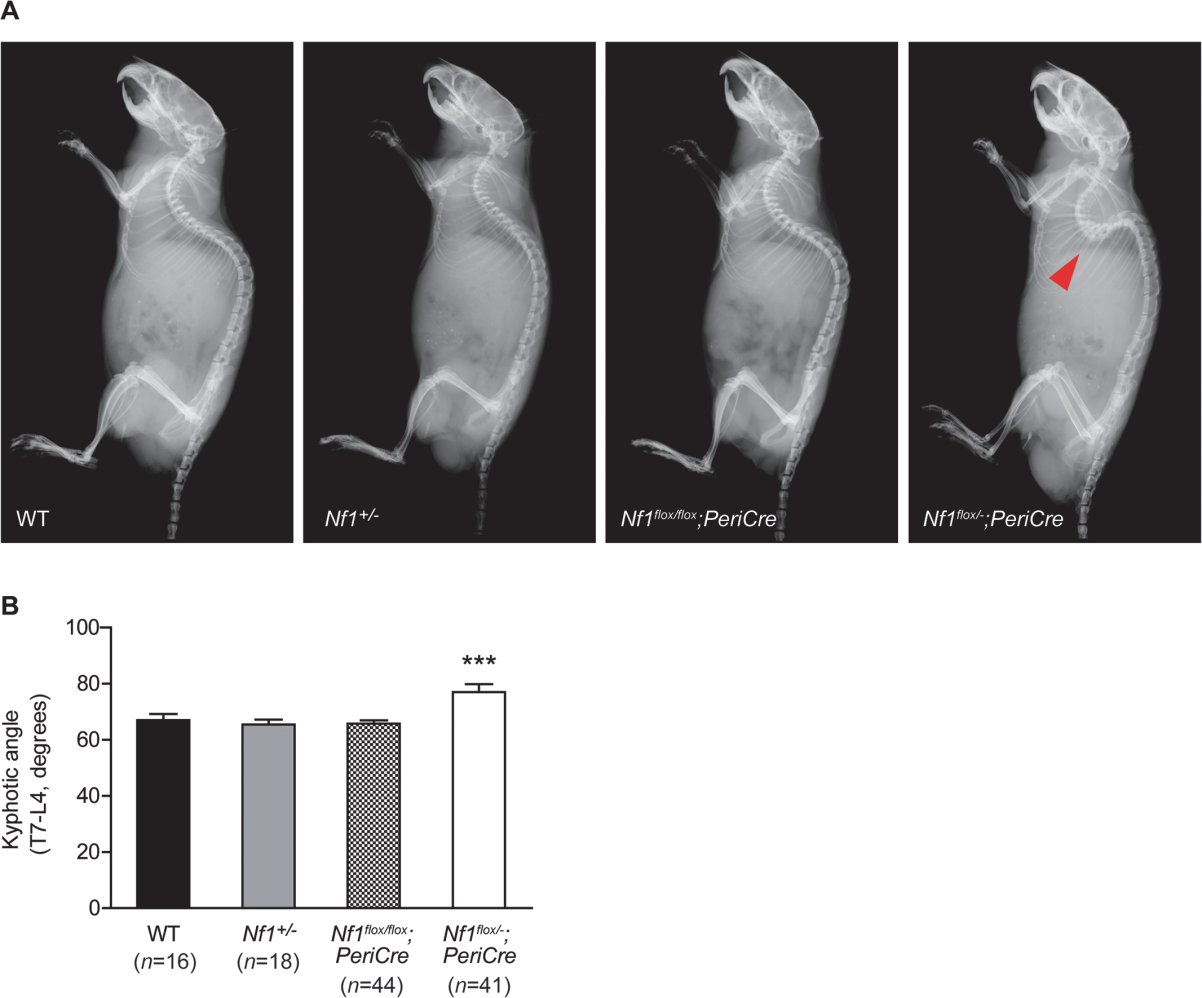
*Nf1* ^flox/−^;*PeriCre* mice exhibit thoracic kyphosis. (**A**) Representative lateral radiographs demonstrate the presence of severe thoracic kyphosis in *Nf1* ^*flox/−*^;*PeriCre* mice as compared to WT, *Nf1* ^*+/−*^, and *Nf1* ^*flox/flox*^;*PeriCre* control animals. (**B**) The mean kyphotic angle was measured using the Cobb technique as defined by the intersection of lines drawn parallel to the rostral T7 and caudal L4 vertebral endplates. Error bars represent the standard error of the mean (SEM). ***P < 0.001 comparing *Nf1* ^*flox/−*^;*PeriCre* mice versus other genotypes.

Kyphosis presents as an exaggerated rounding of the spine resulting in a “hunchback” deformity. When we examined lateral radiographs acquired from *Nf1* ^*flox/−*^;*PeriCre* mice, marked thoracic kyphosis was apparent as compared to the other control strains (Fig. 2A). To further quantify the severity of kyphotic deformity in *Nf1* ^*flox/−*^;*PeriCre* mice, the kyphotic angle from T7-L4 was measured using an adapted Cobb method for mice [46]. A statistically significant increase (P < 0.001) in the mean thoracic angle was observed in *Nf1* ^*flox/−*^;*PeriCre* mice as compared to WT, *Nf1* ^*+/−*^, and *Nf1* ^*flox/−*^;*PeriCre* mice (Fig. 2B). By contrast, eight out of 41 experimental animals in the *Nf1* ^*flox/−*^;*PeriCre* group exhibited kyphotic angles of greater than 90 degrees in lateral radiographs, with a maximum of curvature of approximately 127 degrees in one experimental animal. Consistent with these findings, *Nf1* ^*flox/−*^;*Col2*.*3Cre* animals exhibit a similar phenotype (data not shown). In summary, these data indicate that *Nf1* ^*flox/−*^;*PeriCre* and *Nf1* ^*flox/−*^;*Col2*.*3Cre* mouse models recapitulate several of the characteristic osseous features of NF1 associated dystrophic kyphoscoliosis as seen in NF1 patients.

#### Bone mass, microarchitecture, and remodeling are pathologically altered in *Nf1* ^*flox/−*^;*Col2*.*3Cre* mice with dystrophic spinal deformities

**Fig 3:**
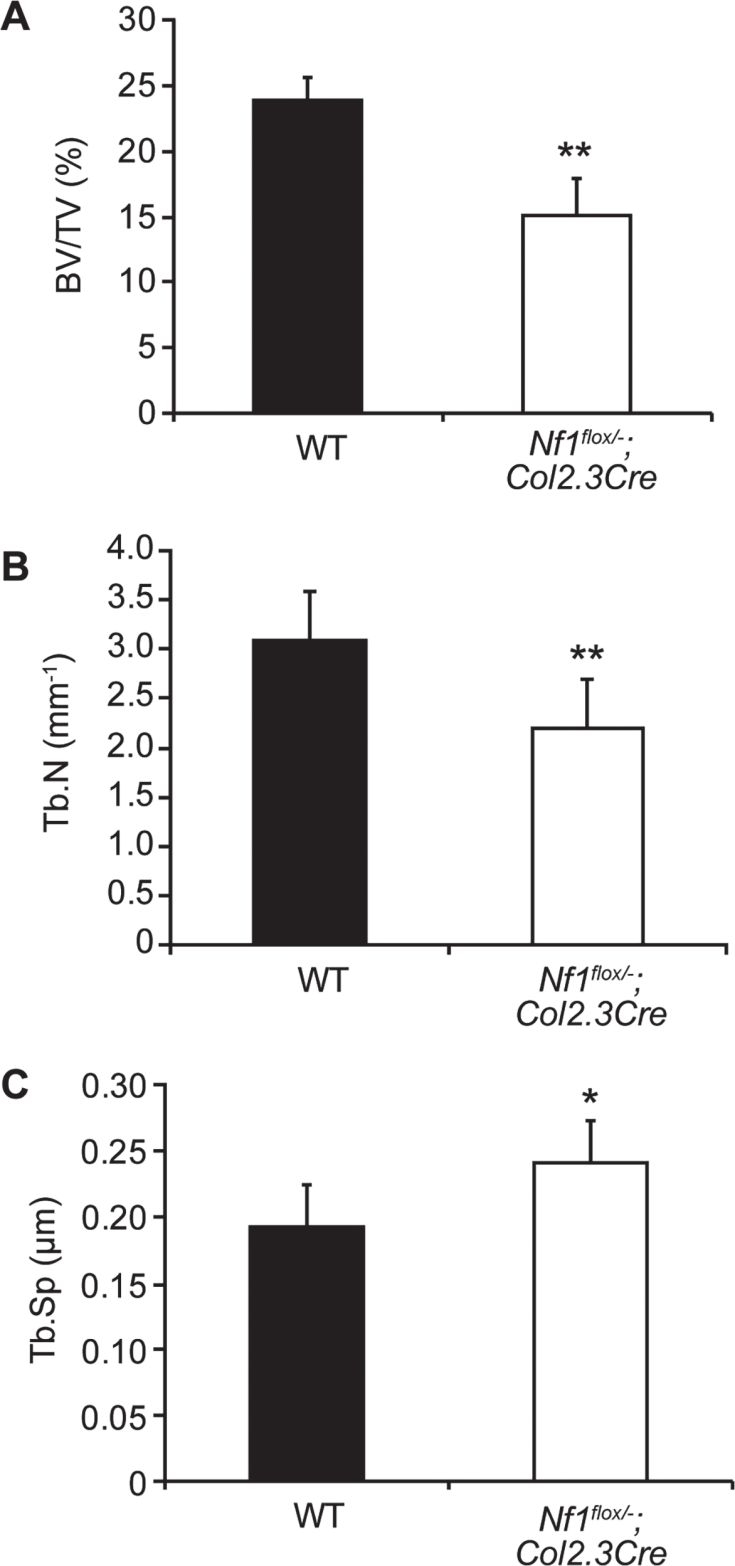
Reduced bone mass and altered trabecular microarchitecture in dystrophic *Nf1* ^flox/−^;*Col2*.*3Cre* vertebrae. (**A**) Percent bone volume (BV/TV, %) was determined following μCT reconstruction of vertebral bone samples. *n* = 5. **P < 0.01 versus WT. (**B**) Trabecular number (Tb.N, mm^−1^) was computed as shown. *n* = 5. **P < 0.01 as compared to WT. (C) Trabecular spacing (Tb.Sp, μm) was calculated by μCT as shown above. *n* = 5. *P < 0.05 as compared to WT.

To further investigate whether dystrophic spinal deformities in the NF1 murine model may be associated with the pathological alterations in bone mass and trabecular microarchitecture, μCT scans were performed on dystrophic vertebrae harvested from *Nf1* ^*flox/−*^;*Col2*.*3Cre* mice. Bone microarchitecture parameters including bone volume fraction (BV/TV, %) and trabecular number (Tb.N, mm^−1^), (Fig. 3A-B) were significantly reduced (P < 0.01) in dystrophic *Nf1* ^*flox/−*^;*Col2*.*3Cre* vertebrae as compared to the corresponding segments from age-matched WT animals controls. Consistent with these findings, trabecular spacing (Tb.Sp, μm) was found to be significantly greater in *Nf1* ^*flox/−*^;*Col2*.*3Cre* vertebrae versus controls (Fig. 3C). Similar phenotypes were observed in *Nf1* ^*flox/−*^;*PeriCre* mice (data not shown) Collectively, these data demonstrate an association between dystrophic spinal deformities in *Nf1* ^*flox/−*^;*PeriCre* and *Nf1* ^*flox/−*^;*Col2*.*3Cre* mice and pathological bone mass and microarchitecture deficits within the dystrophic vertebral segments.

**Fig 4:**
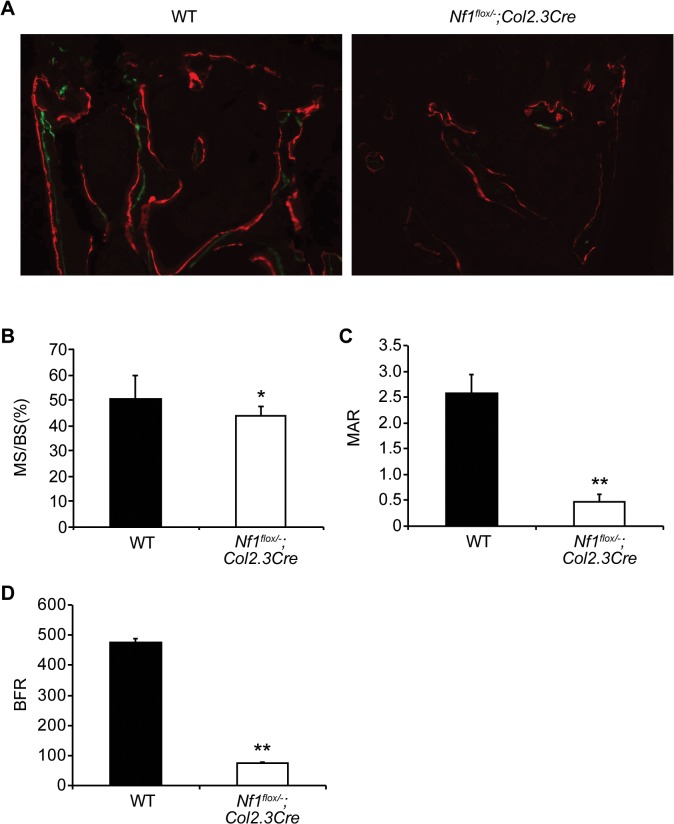
Defective bone remodeling in the lumbar spine of *Nf1* ^flox/−^;*Col2*.*3Cre* mice. Bone remodeling within the L5 vetebral body was examined in WT and *Nf1* ^*flox/−;*^ *Col2*.*3Cre* mice without evidence of dystrophic spinal deformities. (**A**) Representative photomicrographs show calcein and alizarin labeling of L5 vertebrae at 200x magnification from. (**B**) The mineralizing surface (MS/BS, %) was computed as shown. *n* = 5. *P < 0.05 as compared to WT. (**C**) The mineral apposition rate (MAR, μm/day) was calculated according to standard formulas. *n* = 5. **P < 0.01 as compared to WT. (**D**) The bone formation rate (BFR, μm^3^/μm^2^/year) was quantified as shown in the bar graph. *n* = 5. **P < 0.01 versus WT.

Given that bone mass and microarchitecture were significantly impaired in dystrophic vertebrae from *Nf1* ^*flox/−*^;*Col2*.*3Cre* mice, we next sought to ascertain whether these deficits may further be associated with deregulated bone remodeling in the *Nf1* ^*flox/−*^;*Col2*.*3Cre* murine model. To monitor bone formation *in vivo*, two fluorochromes, calcein (20 mg/kg) and alizarin red (20 mg/kg), were administered by intraperitoneal injection 8 and 3 days prior to euthanasia, respectively. Representative photomicrographs illustrate a markedly reduced incorporation of the calcein and alizarin labels within the trabecular bone surface of L5 vertebrae excised from *Nf1* ^*flox/−*^;*Col2*.*3Cre* as compared to WT mice (Fig. 4A). Statistically significant reductions in the active bone-forming surface, or mineralizing surface (MS/BS, %) (Fig. 4B, P < 0.05), and the mineral apposition rate (MAR) (Fig. 4C, P < 0.01), a measure of the rate of radial expansion of new bone were observed in the *Nf1* ^*flox/−*^;*Col2*.*3Cre* L5 vertebrae versus controls. Moreover, the bone formation rate (BFR), an overall measure of bone formation that combines MS/BS and MAR, was dramatically reduced in *Nf1* ^*flox/−*^;*Col2*.*3Cre* mice as compared to WT (Fig. 4D, P <0.01).

#### IVD defects and vertebral fusion in *Nf1* ^*flox/−*^;*PeriCre* and *Col2*.*3Cre* mice

Unexpectedly, radiographic analysis revealed progressive destruction of the intervertebral disc (IVD) space and fusion of adjacent vertebral segments within focal regions of the axial spine in *Nf1* ^*flox/−*^;*PeriCre* and *Col2*.*3Cre* transgenic mice. This phenotype was evident in ∼8.7% (4/46) of *Nf1* ^*flox/flox*^;*PeriCre* animals and ∼36.6% (15/41) of *Nf1* ^*flox/−*^;*PeriCre* mice, but absent in WT and *Nf1* ^*+/−*^ (Table 2). Consistent with these data, *Nf1* ^*flox/flox*^;*Col2*.*3Cre* and *Nf1* ^*flox/−*^;*Col2*.*3Cre* mice exhibit a similar phenotype, with an increased frequency of vertebral fusion in *Nf1* ^*flox/−*^;*Col2*.*3Cre* mice as compared to *Nf1* ^*flox/flox*^;*Col2*.*3Cre* animals.

**Table 2:** Vertebral fusion anomalies in *Nf1* ^*flox/flox*^ and *Nf1* ^*flox/−*^;*PeriCre* and *Col2*.*3* mice.

Genotype	*n*	Fusion Present	% Affected
WT	29	0	0.00
*Nf1* ^*+/−*^	18	0	0.00
*Nf1* ^*flox/flox*^;*PeriCre*	46	(4/46)*	8.70*
*Nf1* ^*flox/−*^;*PeriCre*	41	(15/41)**	36.59**
*Nf1* ^*flox/flox*^;*Col2*.*3Cre*	15	(1/15)^#^	6.67^#^
*Nf1* ^*flox/−*^;*Col2*.*3Cre*	20	(12/20)^##^	60.00^##^

Anterior-posterior and lateral radiographs of WT (n = 29), *Nf1* ^*+/−*^ (n = 18), *Nf1* ^*flox/flox*^;*PeriCre* (n = 46), *Nf1* ^*flox/−*^;*PeriCre* (n = 41), *Nf1* ^*flox/flox*^;*Col2*.*3Cre* (n = 15), and *Nf1* ^*flox/−*^;*Col2*.*3Cre* (n = 20) mice were acquired to screen for the presence of vertebral fusion anomalies. The percentage of mice exhibiting vertebral fusion defects as well as the frequency distribution of the affected segments are summarized in the table and pie chart below.

*Fusion present at L4–5 in all 4 mice

**Fusion present at multiple spinal levels: T13-L1 = 2 mice, L1–2 = 1 mouse, L3–4 = 3 mice, L4–5 = 6 mice, L5–6 = 2 mice, L6-S1 = 1 mouse

#Fusion present at L4–5 in 1 mouse

##Fusion present at multiple spinal levels: T10–12 = 1 mouse, T12–13 = 2 mice, L2–3 = 2 mice, L3–4 = 3 mice, L4–5 = 3 mice, L5–6 = 1 mouse

**Fig 5:**
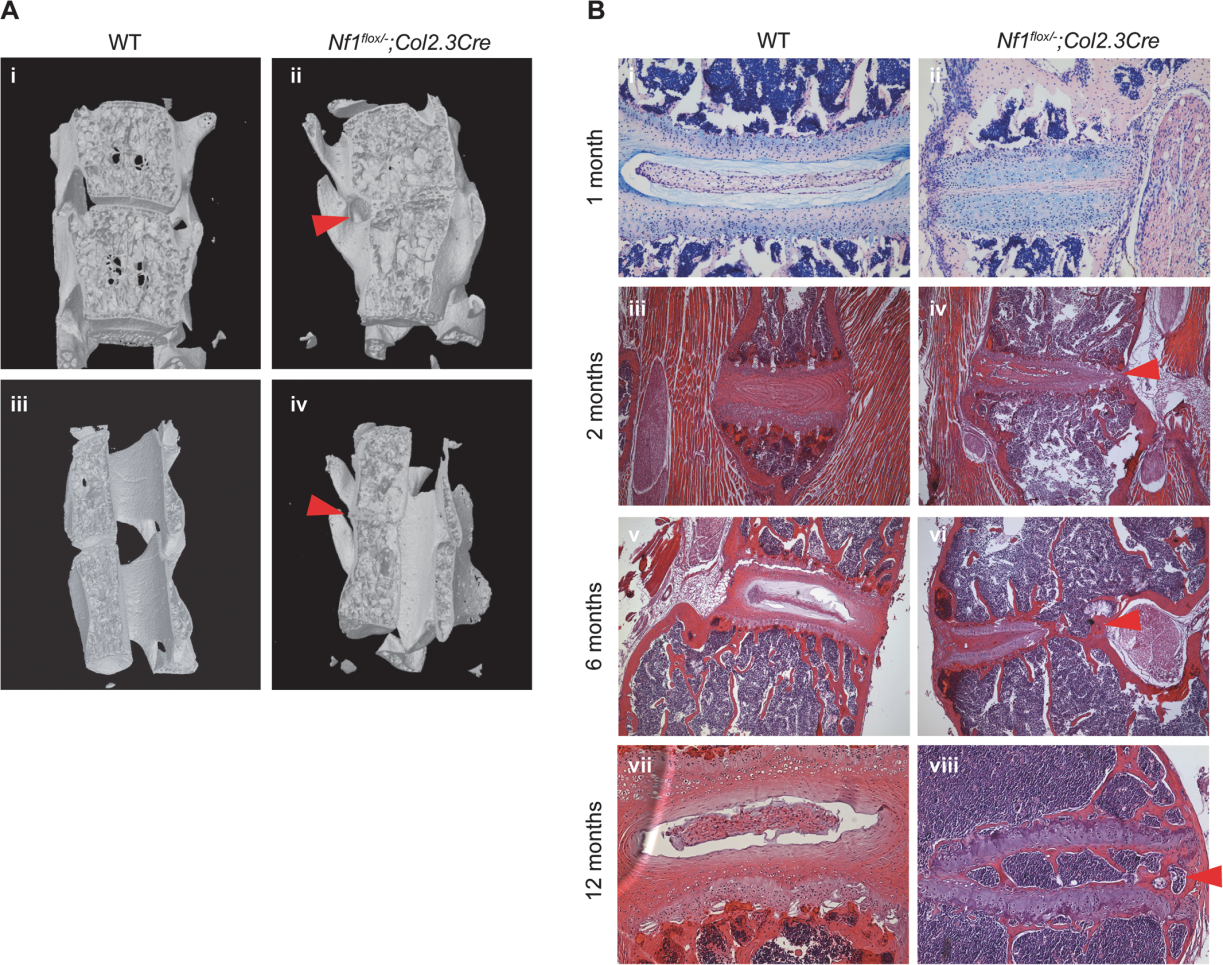
Interarticular fusion in *Nf1* ^flox/−^;*Col2*.*3Cre* mice. (**A**) Representative μCT reconstructions of dystrophic vertebral segments in coronal (top, i and ii) or sagittal (bottom, iii and iv) cross-section. Dysplastic bone growth within the intervertebral disc space has led to interarticular fusion of the dystrophic vertebrae. (**B**) Representative H&E stained histological sections characterize progressive boney dysplasia within the intervertebral disc space of mice at one month (i and ii), two months (iii and iv), six months (v and vi), and 12 months (vii and viii) of age.

Representative sagittal and parasagittal μCT reconstructions further demonstrate vertebral asymmetry and ossification of the IVD space with cancellous bone in *Nf1* ^*flox/−*^;*Col2*.*3Cre* mice (Fig. 5A, ii and iv). Histological sections acquired from mice one, two, six, and 12 months of age illustrate the phenotype in varying stages of progression in *Nf1* ^*flox/−*^;*Col2*.*3Cre* mice (Fig. 5). Atrophy of the AF was evident in *Nf1* ^*flox/−*^;*Col2*.*3Cre* mice beginning from one month of age (Fig. 5B, i-ii). Between two to six months of age, there is evidence of significant degeneration of both the AF and NP with intervertebral fusion beginning from the lateral margins of the disc in *Nf1* ^*flox/−*^;*Col2*.*3Cre* mice as compared to WT control mice (Fig. 5B, iii-vi). Finally, by 12 months of age, complete obliteration of the physiologic IVD architecture is evident in *Nf1* ^*flox/−*^;*Col2*.*3Cre* mice, and the IVD space has been replaced with infiltrating cancellous bone and marrow cells, resulting in interarticular fusion of the adjacent segments (Fig. 5B, vii-viii).

### Human Subjects Data

#### Bone mass and microarchitecture defects in an NF1 patient presenting with dystrophic scoliosis

**Fig 6:**
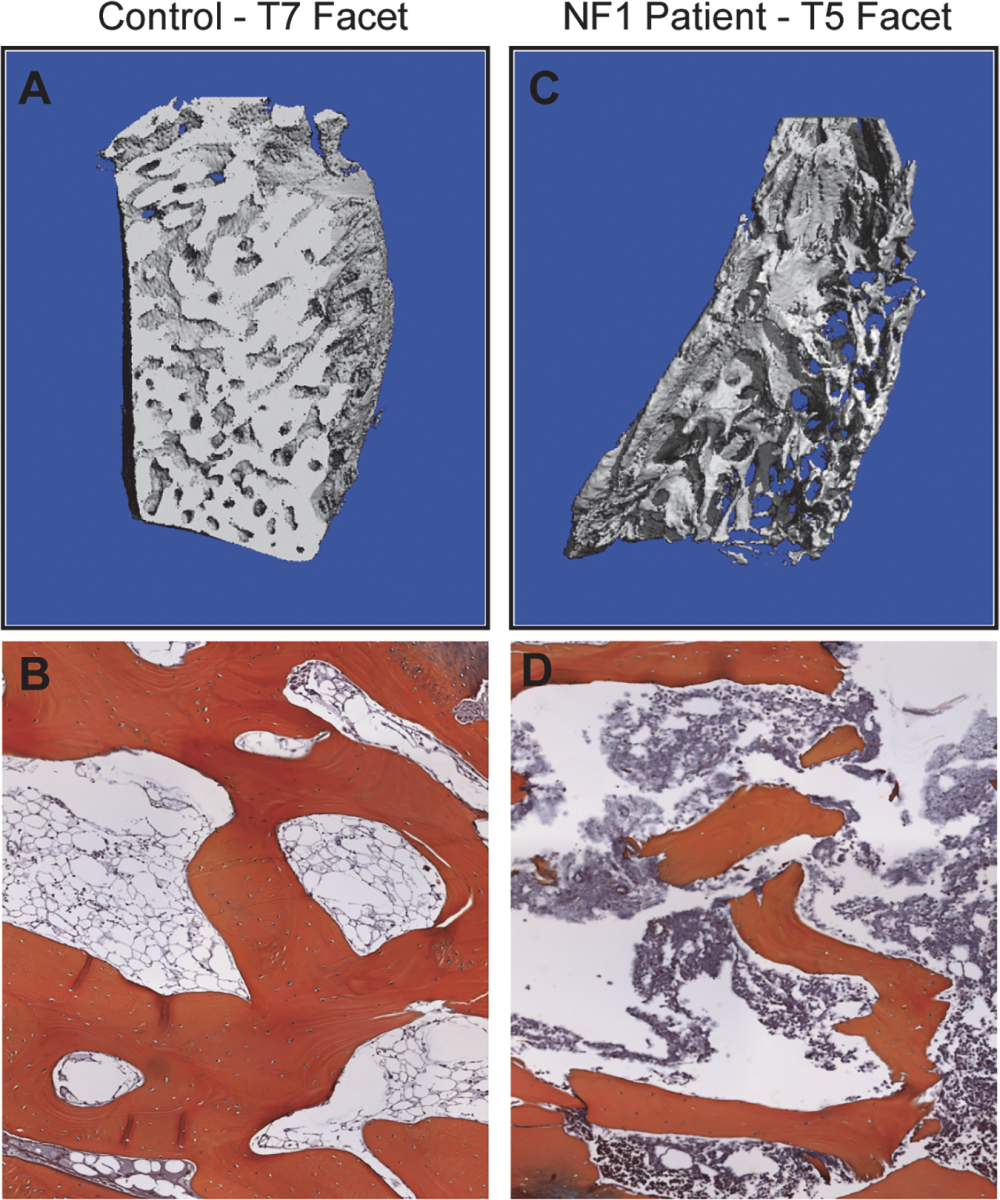
Bone mass and microarchitecture defects in an NF1 patient presenting with dystrophic scoliosis. (**A**) Representative μCT reconstructions of the T7 facet from a sex matched scoliosis patient without NF1 and (**B**) the T5 facet from an NF1 patient with dystrophic scoliosis. (**C**) Representative H&E stained cross sections of vertebral bone of the T7 facet from an age/sex matched scoliosis patient without NF1 and (**D**) the T5 facet from an NF1 patient with dystrophic scoliosis at 40x magnification.

*Nf1* ^*flox/−*^;*Col2*.*3Cre* mice exhibit pathological alterations in bone mass and microarchitecture within the vertebral spine. To investigate whether analogous osseous deficits may also be present in the human condition, we were able to obtain a T5 facet surgically excised from a 10 year old girl with NF1 with dystrophic scoliosis. As a control, we examined the T7 facet from an 18 year old boy without NF1 with idiopathic scoliosis (without NF1) as a control. Representative μCT reconstructions of the excised bone demonstrate markedly reduced bone mass and disrupted trabecular architecture in the T5 facet from the individual with NF1 with dystrophic scoliosis (Fig. 6C, D) as compared to the control sample (Fig. 6A, B). Bone volume fraction (BV/TV), connectivity density (Conn.D., mm^−3^), trabecular number (mm^−1^), and trabecular thickness (Tb.Th, mm) were reduced in the NF1 T5 facet relative to the T7 control; whereas trabecular spacing (Tb.Sp, mm), bone surface/bone volume ratio (BS/BV, mm^−1^), and density anisotropy (DA) were increased in the NF1 T5 facet versus the control (Table 3). In sum, these human data are consistent with the low bone mass phenotype and microarchitectural distortions seen in the *Nf1* ^*flox/−*^;*Col2*.*3Cre* mouse model, however due to the sample size of one and the rarity obtaining of such biopsies, our ability to make more definitive conclusions regarding the clinical phenotype remains limited.

**Table 3:** Quantitative parameters of bone microarchitecture were assessed by μCT in the T5 facet of an NF1 patient with dystrophic scoliosis versus the T7 facet of a control patient with idiopathic scoliosis but without NF1.

Microarchitecture Parameters	Control – T7 Facet	NF1 – T5 Facet
BV/TV	0.6680	0.2992
Conn.D. (mm^−3^)	146.1781	77.1829
Tb.N (mm^−1^)	6.8418	3.0513
Tb.Th (mm)	0.1999	0.1030
Tb.Sp (mm)	0.0969	0.2457
BS/BV (mm^−1^)	10.4599	19.5740
DA	1.3841	1.6518

Bone volume fraction (BV/TV), connectivity density (Conn.D., mm^−3^), trabecular number (Tb.N, mm^−1^), trabecular thickness (Tb.Th mm), trabecular separation (Tb.Sp, mm), bone surface/bone volume ratio (BS/BV, mm^−1^), and density anisotropy (DA) were quantified as shown. Abbreviations: Bone volume fraction (BV/TV), connectivity density (Conn.D.), trabecular number (Tb.N), trabecular thickness (Tb.Th), trabecular spacing (Tb.Sp), bone surface/bone volume (BS/BV), density anisotropy (DA).

#### C6–7 vertebral fusion in a 24 year old female with NF1

**Fig 7:**
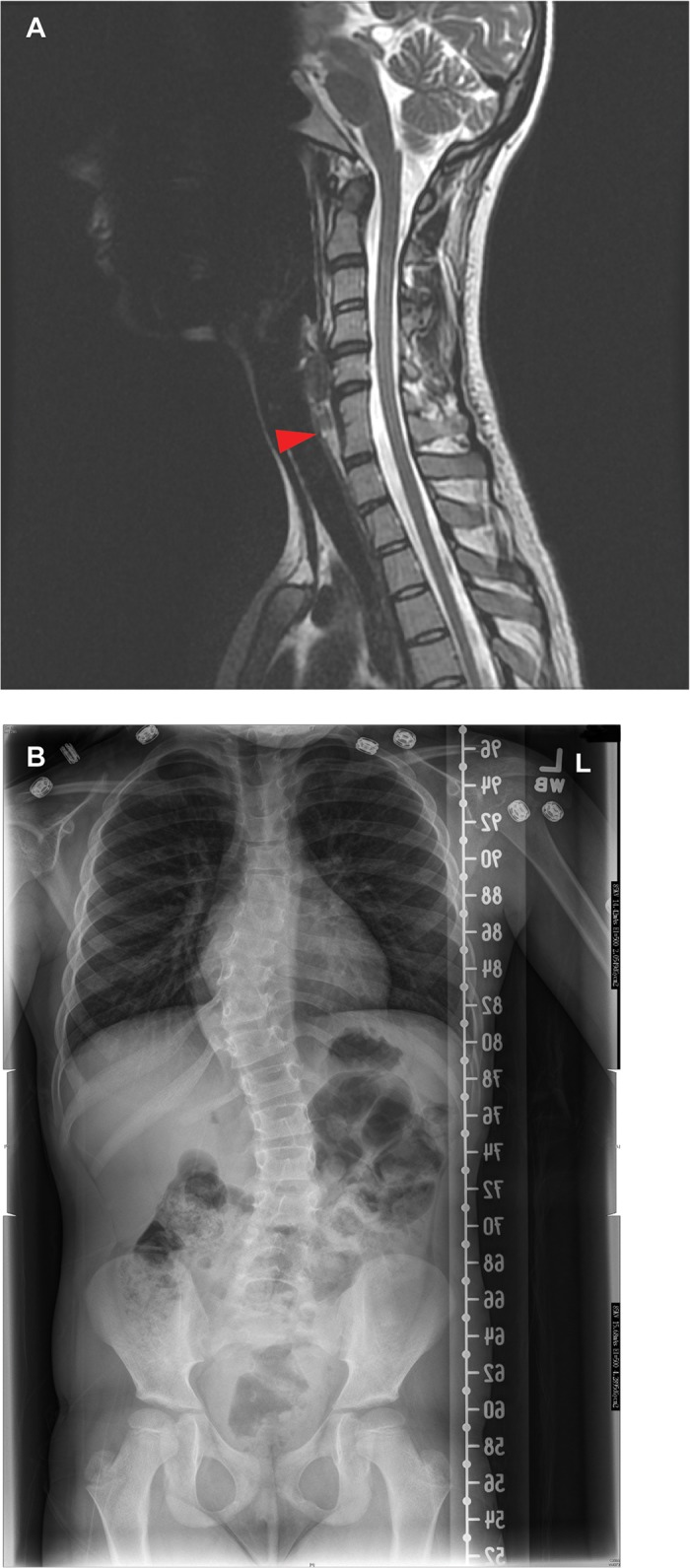
Spinal fusion anomalies in NF1 patients. **(A) Congenital C6–7 vertebral fusion in a 24 year old, female patient with NF1.** MRI shows congenital fusion of the 6th and 7^th^ thoracic vertebrae with an absent disc in a 24 year old woman with a clinical diagnosis of neurofibromatosis type 1 (NF1) based on her history of multiple cafe-au-lait spots, axillary freckling and dermal neurofibromas. **(B) Segmental fusion anomalies at T8-T10 in a 7 year old female NF1 patient with dystrophic scoliosis.** An anterior-posterior standing radiograph of a 7 year old girl with the clinical diagnosis of NF1 (i.e. >6 café au lait macules >0.5cm, axillary and groin freckling, and a first-degree relative with NF1) shows a right thoracic curve measuring 32 degrees between the 8th and 12th thoracic vertebrae and a left thoracolumbar curve measuring 23 degree between the 12th thoracic and 3rd lumbar vertebrae with segmentation and vertebral fusion anomalies at the 8th-10th thoracic vertebrae.

A 24 year old woman with a clinical diagnosis of NF1 (based on her history of multiple cafe-au-lait spots, axillary freckling and cutaneous neurofibromas) presented for consideration of constitutional versus segmental NF1. All skin features were confined to the right arm, upper right back and right axilla. Upon comprehensive review of systems, she was found to have an asymmetric pectus carinatum and radiographic evidence of congenital fusion of the vertebrae at C6/C7 with an absent disc (Fig. 7A).

#### Segmental/fusion anomalies at T8-T10 in 7 year old female with NF1 and dystrophic scoliosis

A 7 year old girl with a clinical diagnosis of NF1 (based on >6 café au lait macules >0.5cm in size, axillary and groin freckling, and a first-degree relative with NF1) presented with asymmetry of the back on examination. The radiology report of standing radiographs of the thoracolumbar spine described a right thoracic curve measuring 32 degrees between the 8th and 12th thoracic vertebrae and a left thoracolumbar curve measuring 23 degree between the 12th thoracic and 3rd lumbar vertebrae with segmentation and fusion anomalies at the 8th-10th thoracic vertebrae (Fig. 7B).

## Discussion

Up to 77 percent of NF1 patients may exhibit scoliosis according to some estimates [25–27]. Surprisingly, however, *Nf1* ^*+/−*^ mice exhibit neither spinal deformities nor any of the other characteristic skeletal deficits seen in the human disease, suggesting that *Nf1* nullizygosity in at least a subset of cell lineages may be required for the genesis of some of these osseous manifestations. Here we demonstrate that conditional biallelic *Nf1* inactivation in mesenchymal stem cells and/or osteoblasts together with a systemic *Nf1* heterozygous genetic background is sufficient to induce characteristic NF1 associated spinal deformities closely reminiscent of that seen in the human disease, including dystrophic and non-dystrophic sharply angulated short-segment curves involving the thoracic and lumbar spine, vertebral rotation, and vertebral wedging.

Unlike *PeriCre*, which drives Cre recombinase expression in both MSCs [41] as well as Schwann cells [44], *Col2*.*3Cre* expression is restricted to mature osteoblasts [45]; and thus, *Nf1* ^*flox/−*^;*Col2*.*3Cre* mice do not develop neurofibromas. The genesis of dystrophic kyphoscoliosis in the *Nf1* ^*flox/−*^;*Col2*.*3Cre* model, together with the associated findings of reduced fractional bone volume, disrupted microarchitecture, and deregulated bone remodeling within the *Nf1* ^*flox/−*^;*Col2*.*3Cre* vertebral spine and the vertebral bone of a human NF1 patient with dystrophic scoliosis are further consistent with the concept that these osseous manifestations may indeed arise as a direct consequence of primary defects in bone cellular function, development, or differentiation, rather than the mass effect of a paraspinal neurofibroma. Given the rarity of obtaining such human bone specimens from the vertebral spine, further studies will be necessary to ascertain whether primary alterations in bone remodeling and microarchitecture are indeed responsible for the observed clinical phenotypes.

The increased penetrance of scoliotic deformities and thoracic kyphosis in *Nf1* ^*flox/−*^;*PeriCre* and *Col2*.*3Cre* mice even as compared to *Nf1* ^*flox/flox*^;*PeriCre* and *Col2*.*3Cre* controls, further suggests that cooperative interactions between *Nf1* nullizygous MSCs/osteoblasts and at least a subset of other *Nf1* heterozygous lineages plays a role in the pathogenesis of these defects in the NF1 murine model. In a series of adoptive bone marrow transfer experiments, we previously established a pivotal role for *Nf1* haploinsufficient hematopoietic cells in the pathogenesis of tibial pseudarthrosis (fracture non-union) in *Nf1* ^*flox/−*^;*PeriCre* and *Col2*.*3Cre* mice [41]. It is plausible that similar heterotypic cellular interactions may be operative in the genesis of dystrophic spinal deformities in the NF1 murine model, although additional experiments are necessary to confirm this hypothesis. In particular, we postulate enhanced bone resorptive activity of *Nf1* haploinsufficient hematopoietic derived osteoclasts to be a likely culprit [34, 36, 37], as we have observed a significant increase in osteoclast activity in the long bones [41] and vertebral spines [41] of *Nf1* ^*flox/−*^;*Col2*.*3Cre* and *Nf1* ^*flox/−*^;*PeriCre* mice. By contrast, a number of previous studies have demonstrated that *Nf1* nullizygous osteoblasts exhibit an impaired capacity to differentiate and mineralize new bone matrix [38, 40]. Thus, enhanced osteolytic activity by *Nf1* ^*+/−*^ osteoclasts, coupled with the inability of *Nf1* deficient osteoblasts to mount an appropriate anabolic response may account for the bone mass, microarchitecture, and remodeling deficits observed in *Nf1* ^*flox/−*^;*Col2*.*3Cre* mice.

Although *Nf1* ^*flox/−*^;*PeriCre* and *Col2*.*3Cre* mice recapitulate many of the hallmark features of dystrophic scoliosis seen in the human disease, we found that the degree of spinal curvature was likely less pronounced in the NF1 murine model than that seen in individuals with NF1 and dystrophic scoliosis where the Cobb angle can reach 70 degrees or greater in severe cases [49]. One potential explanation for this discrepancy may relate to the differing biomechanical circumstances of humans and mice. The vertebrae in quadrupeds do not receive an axial load to maintain balance of the torso, which is the major loading mechanism in bipeds [50]. Consistent with this rationale, it has been demonstrated that scoliotic deformities can be induced with 100% penetrance in rats attaining bipedal posture after undergoing surgical amputation of the tail and forelimbs [51]. It is thus plausible that differing mechanical circumstances between bipeds and quadrupeds may explain why *Nf1* ^*flox/−*^;*PeriCre* and *Col2*.*3Cre* mice do not recapitulate the full extent of lateral spinal curvature seen in NF1 patients with dystrophic scoliosis.

Intriguingly, between 36–60% of *Nf1* ^*flox/−*^;*PeriCre* and *Col2*.*3Cre* mice also exhibit progressive dysplastic deformation of the vertebral bodies, with destruction of the IVD and fusion of adjacent vertebral segments evident by 6–12 months of age. Consistent with these findings, a recent genetic study of mice harboring conditional *Nf1* nullizygous osteochondroprogenitor cells driven by the Col2α1-Cre (collagen, type II, alpha 1) promoter demonstrated congenital IVD formation defects, with improper segmentation of the notochord and impaired proliferation of the nucleus pulposus (NP) accompanied by interarticular vertebral fusion [39]. The authors further note that this phenotype was 100 percent penetrant, with deficits present uniformly at every spinal level. In the present study, we utilized the 3.9kb fragment of the Periostin promoter and the 2.3kb segment of the α(I)-Collagen promoter to achieve a more restricted biallelic ablation of *Nf1* in mesenchymal stem cells and mature osteoblasts, respectively. The reduced penetrance and severity of IVD and fusion defects in the *PeriCre* animal model as compared to the *Col2a1*Cre model published by Wang and colleagues is likely attributable to the robustness of Cre mediated recombination within the MSC compartment, whereby Cre expression in bone marrow derived MSCs cultured from *PeriCre* [41] and *Col2α1Cre* [39] mice appears to be significantly more robust in the latter. Likewise, in contrast to both the *PeriCre* and *Col2a1*Cre animal models, *Col2*.*3Cre* mediated recombination is further restricted to mature osteoblasts alone [45]. Interestingly, in the present study, we observed a significantly increased frequency of IVD and fusion defects in *Nf1* ^*flox/−*^;*PeriCre* and *Col2*.*3Cre* mice as compared to even *Nf1* ^*flox/flox*^;*PeriCre* and *Col2*.*3Cre* controls, suggesting that *Nf1* heterozygosity in surrounding tissues and cell lineages (in the context of *Nf1* nullizygous MSCs and osteoblasts) may play a role in the pathogenesis of these anomalies.

In contrast to the aforementioned murine models, vertebral fusion and IVD defects in the NF1 patient population have rarely been documented in the medical literature. In 1986, Sartoris and colleagues documented one case of presumptive neuropathic arthropathy in a NF1 patient presenting with destruction of the L1-L2 vertebral bodies and intervertebral disk space [52]. A retroperitoneal neurofibroma was identified anterior to the L1-L2 interspace; however, no neurofibromatosis tissue was found within the disk space itself, leaving questions regarding the underlying etiology. Here we present two cases of IVD defects/vertebral fusion in patients with a clinical diagnosis of NF1. The first case involves a 24 year old woman with multiple café-au-lait macules, axillary freckling, dermal neurofibromas, and pectus carinatum presenting with congenital C6–7 vertebral fusion with an absent disc. The second individual was a 7 year old girl with NF1 and dystrophic scoliosis with segmentation and fusion anomalies at the 8th-10th thoracic vertebrae. The paucity of interarticular vertebral fusion and IVD defects reported in the medical literature likely reflects inherent differences between murine models of the disease versus the human condition. Whereas biallelic Cre-mediated *Nf1* recombination within osteoblasts and/or osteoprogenitor cells occurs homogenously in animal models, clinically such losses of *NF1* heterozygosity are likely to be relatively rare events, occurring only within focal regions of the bone or sometimes not at all within any given patient. Nonetheless, it is also possible that these deficits have been overlooked or underreported in NF1 patients. Future retrospective radiographic analysis of larger patient cohorts may help clarify whether this phenotype may have been previously overlooked or underreported in the NF1 patient population.
